# Combined Radiotherapy and Hyperthermia: A Systematic Review of Immunological Synergies for Amplifying Radiation-Induced Abscopal Effects

**DOI:** 10.3390/cancers16213656

**Published:** 2024-10-30

**Authors:** Loïc Van Dieren, Tom Quisenaerts, Mackenzie Licata, Arnaud Beddok, Alexandre G. Lellouch, Dirk Ysebaert, Vera Saldien, Marc Peeters, Ivana Gorbaslieva

**Affiliations:** 1 Vascularized Composite Allotransplantation Laboratory, Center for Transplantation Sciences, Massachusetts General Hospital, Harvard Medical School, Boston, MA 02114, USA; 2 Faculty of Medicine and Health Sciences, University of Antwerp, 2610 Wilrijk, Belgium; 3 Boston Children’s Hospital, Boston, MA 02115, USA; 4 Institut Godinot, Radiation Oncology Department, 85054 Reims, France; 5 GCMI, Massachusetts General Hospital, Harvard Medical School, Boston, MA 02114, USA; 6 Department of Hepatobiliary, Transplantation and Endocrine Surgery, University Hospital of Antwerp, 2650 Edegem, Belgium; 7 Department of Anesthesiology, University Hospital of Antwerp, 2650 Edegem, Belgium; 8 Department of Oncology, University Hospital of Antwerp, 2650 Edegem, Belgium

**Keywords:** cancer, hyperthermia, immune system, metastasis, radiotherapy

## Abstract

Introduction: The abscopal effect is a systemic immune response characterized by metastases regression at sites distant from the irradiated lesion. This systematic review aims to explore the immunological mechanisms of action underlying the abscopal effect and to investigate how hyperthermia (HT) can increase the chances of radiotherapy (RT) triggering systemic anti-tumor immune responses. Methods: This review is created in accordance with the PRISMA guidelines. Results and Conclusion: HT and RT have both complementary and synergistic immunological effects. Both methods trigger danger signal release, promoting cytokine and chemokine secretion, which increases T-cell infiltration and facilitates cell death. Both treatments upregulate extracellular tumor HSP70, which could amplify DAMP recognition by macrophages and DCs, leading to stronger tumor antigen presentation and CTL-mediated immune responses. Additionally, the combined increase in cell adhesion molecules (VCAM-1, ICAM-1, E-selectin, L-selectin) could enhance leukocyte adhesion to tumors, improving lymphocyte trafficking and boosting systemic anti-tumor effects. Lastly, HT causes vasodilation and improves blood flow, which might exacerbate those distant effects. We suggest the combination of local radiotherapy with fever-range whole-body hyperthermia to optimally enhance the chances of triggering the abscopal effect mediated by the immune system.

## Simple Summary

Cancer treatments like radiotherapy can sometimes cause tumors to shrink not only in the treated area but also in other parts of the body. This phenomenon, called the abscopal effect, happens when the immune system is triggered to attack tumors at distant sites. This review explores how heating the body (hyperthermia) alongside radiotherapy may improve the likelihood of this effect. Both treatments work by sending “danger” signals to the immune system, helping it recognize and attack cancer cells. Radiotherapy and hyperthermia together increase immune activity by releasing specific proteins and improving blood flow to the tumor, making it easier for immune cells to reach and attack cancer cells. This combination may offer a promising way to boost the body’s natural defenses against cancer, helping more people experience widespread tumor shrinkage and potentially improving cancer treatment outcomes.

## 1. Introduction

Metastasis, the phenomenon in which primary tumors spread to surrounding tissues and distant organs, is the leading cause of cancer mortality and morbidity [1]. Despite the application of conventional therapies such as radiotherapy (RT), chemotherapy (CT), immunotherapy (IT) and surgery in clinical settings, further treatment development and optimization is required to mitigate metastasis progression. Treating metastasis in cancer patients, in particular, continues to challenge researchers. Hyperthermia (HT), a relatively novel approach to treating cancer metastasis, has the capacity to improve related morbidity and mortality rates [2]. 

HT involves using an external heat source to elevate tissue temperatures. Treatment temperatures range from 38 °C (fever-range HT) to 80 °C (thermal ablation) and beyond, for a defined period of time [3]. HT can be applied locally, regionally or to the whole body (WBHT). Local HT involves applying heat to a restricted area, such as a solid tumor ,while regional hyperthermia is applied to a relatively larger area, such as a limb. WBHT, in contrast, is when the entire body is heated, and this is the primary method currently used to treat metastatic cancer [4]. However, new research indicates that, by inducing an abscopal effect, local or regional HT can alternatively be used to treat metastatic cancer [5,6]. As first described in the RT field, the abscopal effect reflects the regression of non-irradiated metastatic lesions at a distance from the primary site of irradiation [7]. The underlying mechanisms explaining this effect are not yet understood [8]. However, in mouse studies, the observations support the abscopal effect immune hypothesis; no abscopal effect was observed in immune-deficient mice [9]. Ionizing radiation stimulates the release of damage-associated molecular patterns (DAMPs) and inflammatory cytokines, changes the tumor microenvironment (TME), induces immunologic cell death, and activates immune cells [10]. Under the appropriate conditions, the above-mentioned effects can trigger systemic anti-tumor immunity effects [11], which leads to the abscopal effect. 

HT induces some of these effects as well. Locally heated tumors between 39 °C and 45 °C can induce anti-tumor immune responses. HT enables tumor cells to stimulate the immune system through the increased expression of MHC-I; the release of heat-shock proteins (HSPs); the activation of natural killer (NK) cells, cytotoxic T-cells (CTL’s), and dendritic cells (DC’s); and improved immune cell trafficking [12]. The use of RT to trigger the abscopal effect has been widely researched, and current research is focusing on adding IT to RT to boost the abscopal effect [13]. The effects of HT on the immune system have not yet been thoroughly researched. The objective of this article is to enhance our comprehension of the immunological processes activated by HT and RT and to uncover potential synergies between the two. By describing the pro-tumorigenic and anti-tumorigenic mechanisms associated with the abscopal effect, we aim to investigate how radiotherapy can leverage whole-body hyperthermia (WBHT) to augment its potential for inducing systemic anti-tumor immune responses in metastatic cancer.

## 2. Methods

This review was performed in accordance with the PRISMA (Preferred Reporting Items for Systematic Reviews and Meta-Analyses) guidelines and has not been registered. We systematically searched Web of Science and Medline (PubMed) in February 2022, using following MeSH terms: “Radiotherapy”, “Hyperthermia, Induced”, and “Immune System Phenomena”. Two search queries were performed: ((“Radiotherapy”[Mesh]) AND “Immune System Phenomena”[Mesh] AND ((review[Filter] OR systematic review[Filter]) AND (2017:2022[pdat]))) and (“Hyperthermia, Induced”[Mesh]) AND “Immune System Phenomena”[Mesh] AND ((review[Filter] OR systematic review[Filter]) AND (2000:2022[pdat])). To collect high-quality evidence, we decided to only include reviews and systematic reviews. 

Next, two authors performed a primary appraisal based on titles and abstracts. The title or abstract must mention “radiotherapy” or “hyperthermia” and “immune*”. We excluded articles discussing the combination of RT and other treatment modalities like IT and CT, articles focusing on other diseases than cancer, and articles describing therapeutic strategies other than RT or HT. We also decided to exclude articles focusing on thermal ablation. In cases of discordance, a third author decided whether to in- or exclude the article. Next, three reviewers performed data collection. Each reviewer collected data from two-thirds of the articles. Thus, the data from each article were collected twice. The current systematic review is written in accordance with the PRISMA guidelines. 

## 3. Results

**Figure 1:**
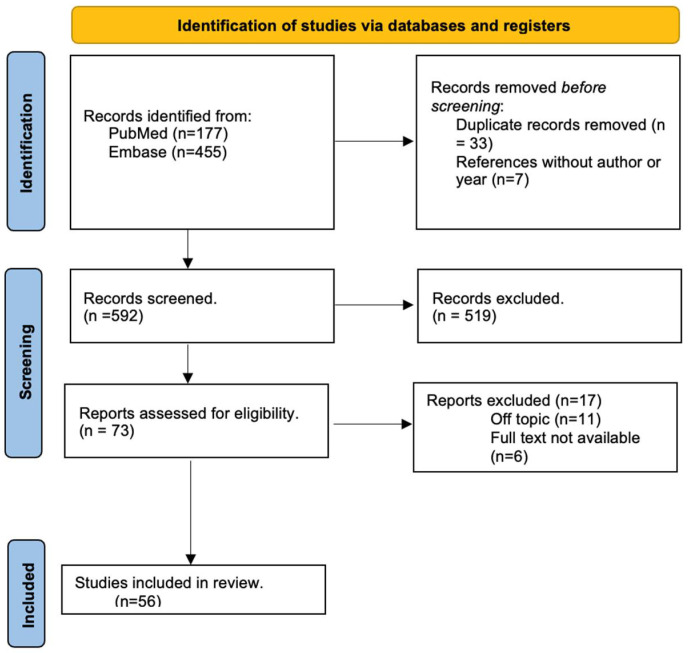
Prisma flowchart of the included studies.

Six-hundred thirty-two articles relating to the immune-system phenomena of RT and HT were ultimately identified. After the primary selection, in which articles were filtered out based on missing key words in the title and abstract, 73 articles remained. Thus, 56 articles were finally included in this manuscript. The 17 post hoc-excluded articles were off-topic (Figure 1). 

### 3.1. Radiotherapy

Because immune cells are highly radiosensitive, it was initially hypothesized that RT is mainly immunosuppressive [14]. However, a recent paradigm shift indicates that RT also reprograms our immune system to attack tumor cells. This is likely mediated by immunogenic cell death (ICD), which is responsible for the bystander and abscopal effects [15,16]. 

#### 3.1.1. Immunogenic Cell Death

**Figure 2:**
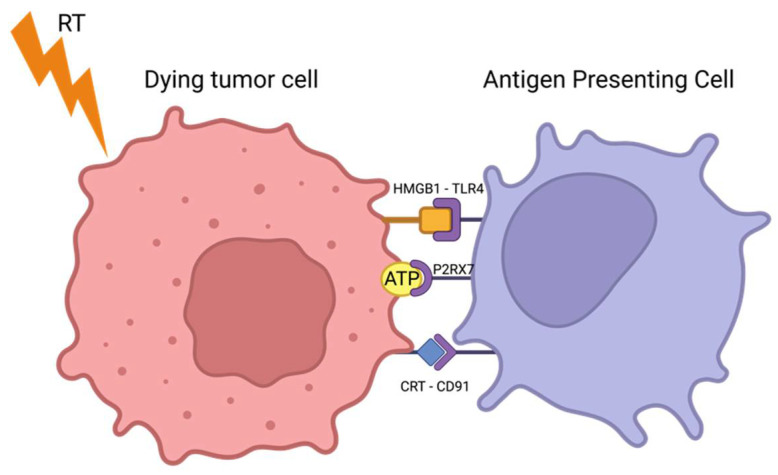
DAMP release induced by RT and its subsequent recognition by an immune cell. RT (indicated by the lightning bolt) triggers the presentation of ATP, calreticulin (CRT) and high-mobility group box 1 (HMG1) from the tumor cell. The released DAMPs bind to purinergic receptors on the surface of the immune cell, initiating a signaling cascade that enhances the immune cell’s ability to recognize and respond to the tumor cell. This interaction is crucial for the activation and recruitment of immune cells to the tumor microenvironment, thereby boosting the anti-tumor immune response.

In contrast to apoptosis, ICD is associated with antigen release from the dying tumor cells and leads to an immune reaction resulting from the antigen presentation and activation of subsequent immune cells [17,18]. The expression of DAMPs by irradiated tumor cells plays an important role in this immune-mediated mechanism [19]. After the exposure of the tumor cells to ionizing radiation (IR), multiple DAMPs are expressed, secreted or translocated: (i) the exposure of calreticulin (CRT) on the cell surface acts as an “eat me” signal, which stimulates phagocytosis by macrophages and immature DCs; (ii) ATP activates myeloid cells and recruits immature DCs, and it is also involved in T-cell priming by secreting IL-1b; (iii) high-mobility-group-box 1 (HMGB1) is released by dead cells and interacts with toll-like receptor 4 (TLR4), thus promoting antigen presentation; and (iv) heat-shock protein 70 (HSP70), translocated from the cytoplasm, activates monocytes, macrophages and DCs. It directly activates and triggers the cytolytic activity of NK-cells [20,21,22,23,24]. Altogether, these DAMPs polarize and activate DCs and macrophages through engagement with TLRs present on their surface [25,26] (Figure 2). 

Upon activation, DCs and macrophages take up tumor-associated antigens (TAAs) in the TME. They travel through lymphatic vessels and cross-present the TAAs in the tumor-draining lymph nodes to major histocompatibility complex I or II (MHCI or MHCII) molecules [27]. In the lymph nodes, CTLs and T-helper cells (Th-cells) are primarily activated and undergo differentiation upon antigen presentation, while NK-cells may receive signals that enhance their activation and function. Upon release into the blood circulation, the activated immune cells induce an anti-tumor reaction. RT-activated T-cells also produce TNF-α, which eliminates myeloid-derived suppressor cells (MDSCs) locally and systemically [21]. The RT-induced immune cells can return to the primary tumor, but they can also travel to distant metastases originating from the primary tumors eliciting abscopal effects. It is important to note that inclusion of the draining lymph node in the irradiated field has a deleterious effect on this process [28,29]. This is likely caused by locally depleting the radiosensitive immune cells.

#### 3.1.2. The cGAS/STING Pathway

**Figure 3:**
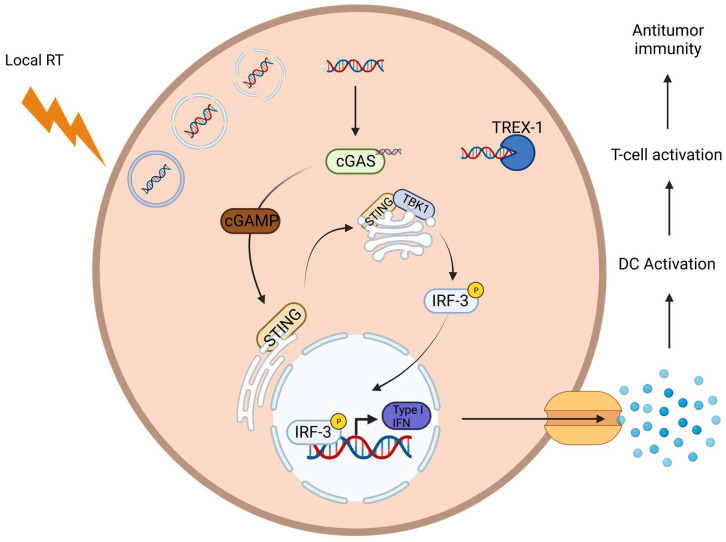
Activation of the cGAS-STING pathway by radiation therapy. Radiation therapy (indicated by the lightning bolt) causes the release of cytosolic DNA within the cell. This DNA is detected by the cyclic GMP-AMP synthase (cGAS), which then produces cyclic GMP-AMP (cGAMP). cGAMP binds to the stimulator of interferon genes (STING) on the endoplasmic reticulum, leading to its activation. Activated STING recruits and activates TANK-binding kinase 1 (TBK1), which in turn phosphorylates interferon regulatory factor 3 (IRF-3). Phosphorylated IRF-3 translocates to the nucleus, where it promotes the production of type I interferons (IFNs). These interferons are then secreted from the cell, enhancing the immune response by promoting the recruitment and activation of immune cells. Additionally, TREX1, a DNA exonuclease that degrades cytosolic DNA regulates this pathway.

Another RT-induced, immune-dependent mechanism involves the cGAS/STING pathway. In this process, double-stranded DNA (dsDNA) condenses into micronuclei as a result of irradiation-induced double-strand breaks (DSBs). However, the membranes of these micronuclei are prone to rupture, leading to the leakage of their contents into the cytosol. The dsDNA is then detected by cytosolic DNA sensors: the cyclic GMP-AMP synthases (cGAS) [30]. cGAS catalyzes cGAMP, which binds to STING. STING then translocates from the endoplasmic reticulum to the Golgi-apparatus, where it binds to TBK1. This phosphorylates and activates IRF3, which binds to the STING-TBK1 complex. IRF3 then dislocates from the complex and translocates to the nucleus, where it stimulates type-I IFN (IFNa/ß) production [9]. cGAS/STING-mediated type-I IFN production stimulates antigen presentation in the lymph nodes by DC recruitment and the activation/priming of T-cells. Additionally, the induction of IFN-I by irradiation increases the levels of CXCR3 (a chemokine receptor), which recruits CTLs to the TME [31]. Activated T-cells secrete type-II IFN (IFNγ) and enhance MHC-I expression on tumor cells. Additionally, IFNγ modulates the tumor vasculature to improve T-cell trafficking and enhances T-cell recruitment by stimulating chemokine secretion [32]. Overall, the cGAS/STING pathway supports the immune system-induced abscopal effects by increasing immune recognition, improving T-cell trafficking, enhancing T-cell recruitment, and stimulating chemokine secretion [32,33]. However, it is important to note that doses higher than three fractions of 8 Gy activate DNases like TREX1, which degrades cytoplasmic DNA and prevents the adequate activation of the cGAS/STING pathway. It also prevents DC recruitment and the priming of T-cells and abrogates the immune-mediated systemic response outside of the irradiated field [27,33,34] (Figure 3).

#### 3.1.3. Effects of RT on the TME

RT also modulates the TME [35]. RT stimulates the secretion of CXCL16, CXCL9 and CXCL10 (inflammatory chemokines) and the expression of ICAM-1, VCAM-1 and E-selectin (cell adhesion molecules); endothelial activation is triggered by TNF-α and IL-6 [36]. These cell adhesion molecules are critical for leukocyte migration from the bloodstream to the tumor [37]. The upregulation of these molecules facilitates the recruitment of anti-tumor T-cells and triggers an immune response accompanied by the release of pro-inflammatory molecules like IL-1, IL-2, IL-6, IL-8, IL-12, VEGF, EGFR, IFN-α, IFN-ß and TNF-α. All these cascade reactions lead to the pro-inflammatory microenvironment, which stimulates DAMP release [38]. 

#### 3.1.4. Exosomes

There is still discussion about the mechanisms through which the cells communicate to induce the abscopal effect. Exosomes might play an important role in mediating the immunomodulatory effects of RT. They can transport immunostimulatory as well as immunosuppressive molecules [38]. Unirradiated bystander cells have the ability to take up these exosomes, including their content, such as DNA, microRNA and proteins. The uptake of the exosomes can lead to altering multiple pathways that may promote immune stimulation. For instance, by transferring TAAs, the anti-tumor response could be amplified and cause an increase in antigen presentation, immune cell recruitment and cytotoxic activity [39].

#### 3.1.5. RT Modalities and Dosage

Compared to low-radiation doses, higher doses induce significantly greater anti-tumor immune responses [40]. The increased effect is mediated by a higher presentation of tumor-associated antigens, the upregulation of MHC-I, and an increased presentation to CD8+ T-cells [25,41]. In addition to radiation dose, the immunomodulating properties of RT rely on fractionation [42]. Studies show that larger, single-dose regimens are superior, if not at least equivalent to those delivered in multiple lower-dose fractions [43]. Again, this effect is mediated by the infiltration of CD8+ T-cells. However, when analyzing the effects on distant metastases, Diegeler et al. suggests that high-dose-per-fraction (≥6–8 Gy) regimes result in better in situ vaccination than single-dose therapy. Therefore, they conclude that high-dose fractionated therapy shows the most systematically immunogenic effects [25]. In contrast, other authors state that the hypofractionated regimens stimulate the immune system the most [20,33,44,45]. Low-dose RT has been described to increase polarization towards the anti-tumor M1-macrophages, decrease CAFs, and reduce TGF-ß. However, compared to high doses, low doses of RT (<5 Gy) induce apoptotic cell death, which is not immunogenic. Additionally, DCs take up apoptotic bodies, which prevents them from maturing and exerts a tolerogenic effect [45]. In fact, high-dose RT is associated with a higher priming of T-cells and antigen release and low-dose RT creates an immune-favorable TME. These observations have led to the RadScopal TM technique, which is an approach that combines high-dose RT for the primary tumor with low-dose RT for the metastatic lesions to favor anti-tumor immune responses [46]. Overall, the ability of RT to trigger in situ vaccination is mainly dependent on the radiation dose, the regimen involved and the pre-existing TME [20].

#### 3.1.6. Concluding Remarks

Overall, the release of TAAs and their recognition by antigen-presenting cells (APCs), the activation of type-I IFN by the cGAS/STING pathway, and the formation of a pro-inflammatory microenvironment lead to the activation of the innate and adaptive immune system. The result of the cascade reaction leads to systemic anti-tumor effects, but unfortunately, the abscopal effect is only observed in a few cases [47]. The conversion of the TME into a pro-inflammatory state remains transient due to the anti-inflammatory response empowered by cancer cells and their immunosuppressive TME [48]. 

### 3.2. Hyperthermia

#### 3.2.1. Immune Cell Trafficking

HT increases RT efficacy based on higher cell-killing effect [49]. Heat prevents cell recovery following sublethal cell damage by inhibiting the repolymerization step during damage base repair. Consequently, heat increases DNA fragmentation [4,50]. Besides increased cell killing, HT also sensitizes hypoxic tumor areas—which are known for their resistance to RT [51]—by increased blood flow and oxygenation [52]. The combination of hypoxia and moderate HT also causes macrophages to produce superoxides, which reduces VEGF expression and suppresses HIF-1 expression [51]. Better tumor perfusion is also associated with better immune cell trafficking. This facilitates the traveling of APCs between the tumor and the draining lymph nodes [6]. 

Improved tumor perfusion is associated with better immune cell trafficking. In addition, increased cell adhesion molecules also contribute to the immune cell trafficking process [6]. Under normal conditions, immune cells are excluded from the TME due to the downregulation of ICAM-1 and integrins [53]. Local and WBHT can increase the lymphocyte trafficking to the tumor area across specialized blood vessels called high-endothelial venules (HEVs). Fever-range temperature (38–42 °C) enhances the endothelial expression of ICAM-1, L-selectin and CCL21 [53,54]. This increase is absent in normal vasculature and leads to the selective delivery of cytotoxic T-cells to the tumor area. Additionally, the increased expression of a4 b7 integrin on lymphocytes allows them to specifically interact with HEVs. This is caused by fever-range HT and increases homing to secondary lymphoid tissue [55]. In summary, the increased expression of ICAM-1, L-selectin, CCL21 and a4b7 boosts the chances of antigen specific T-cells encountering APCs in the lymph nodes [54] and creates a TME enriched in leukocytes. Both mechanisms enhance the likelihood of triggering a systemic anti-tumor immune response and enhance the tumoricidal effects of the immune system [56].

#### 3.2.2. HT-Triggered Immunogenicity

Baronzio et al. states that HT increases antigen presentation by two mechanisms: HT increases (i) the immunogenicity of tumor cells and (ii) the production of HSPs and co-stimulatory molecules [53]. Some studies report an increased expression of TAAs after exposure to heat, but other studies report a decrease in immunogenicity due to a downregulation of TAAs or MHC-I expression [51,53]. Generally, temperatures above 45 °C are known to reduce MHC-I expression, whereas temperatures below 45 °C do not affect MHC-I surface expression [51]. Extracellular HSP70 is significantly increased after the exposure of tumor cells to HT and RT [4]; it participates in the migration and homing of DCs and facilitates antigen presentation to CD8+ T-cells [57]. Moreover, HT upregulates TLR2 and TLR4 on DCs and macrophages [4]; stimulates DC to produce IL-12 and TNF-α (Th1 cell polarizing cytokines) [53,55]; increases FasL expression on cytolytic T-cells [51]; fosters the cross-priming of TAAs to DCs [4]; increases the migration of DCs to draining LNs [6]; and increases the DC expression of MHC-I and II, CD80 and CD86 [55]. Hence, fever-range temperatures enhance the ability of DCs to cross-present tumor antigens to T-cells, which leads to their activation and priming against the tumor. 

#### 3.2.3. Heat-Shock Proteins

As a result of heat exposure, HSF is activated and produces multiple HSPs within minutes [50]. Their expression increases linearly until a certain temperature threshold—this is dependent on cell type. Beyond this threshold, HSP synthesis is inhibited, and cell death occurs because of thermotolerance [53]. Now, HSPs are also recognized as immunostimulants when they are complexed with peptides and expressed on the tumor cell surface or are released extracellularly [58]. They act as DAMPs that may be recognized by the innate immune system and cause the activation of DCs and macrophages. This is accompanied by an inflammatory cytokine cascade that comprises IL-6, IL-12 and TNF-α (these cytokines help in the maturation of DCs towards Th1-helper cells) [53,54]. Upon recognition of the HSP-bound peptides, APCs can produce antigen-specific CTL responses. This cross-presentation is presumably more rapid and efficient compared to free antigens, which suggests an involvement of the adaptive immune system [54]. HSP70 is recognized as being the most immunogenic heat-shock protein, and its surface expression is increased after the exposure of HT, which correlates with an enhanced lytic capacity of NK-cells [6,59]. Extracellular HSP70 is thought to be released through the involvement of exosomes [60]: Linder et al.]. This may further enhance the migratory and lytic capacity of NK-cells [53]. HSP70-containing exosomes also attract and activate DCs and T-cells through the following chemokines: CCL2, CCL3, CCL4, CCL5 and CCL20 [4,61]. HSP60 and HSP90 are also involved in T-cell activation upon antigen presentation through the production of IFN-γ [54,62]. 

In summary, extracellular HSP70 complexes—released after thermal stress—act as a danger signal for the immune system. The exosome-mediated release of cyto- and chemokines attract and activate DCs, which take up TAAs and present them in tumor-draining lymph nodes, where CTLs are activated and primed and may exert their systemic anti-tumor functions. Lastly, HT pre-associates the components of the T-cell receptor (TCR), which lowers the threshold for T-cell signaling and effector T-cell differentiation [55].

#### 3.2.4. Natural Killer Cells

NK-cells play a major role in HT efficacy. They are responsible for eliminating tumor cells that evade the adaptive immune system by downregulating the MHC-I class molecules on their surface. By depleting the MHC-I molecules on their surface, tumor cells render themselves invisible to CTLs, but a specific subset of NK-cells can target cells lacking MHC expression [51]. Essentially, NK-cell cytotoxicity is enhanced after mild HT (<40 °C), which causes the clustering of NKG2D stimulatory receptors on the NK-cell surface and an increased expression of the stimulatory ligands, HSP70 and MICA, on the tumor cell surface. However, the enhanced cytotoxicity of NK-cells is reversed at higher temperatures [50,63]. HT also enhances the anti-tumor properties of granulocytes [4], and fever-range HT recruits neutrophils to the tumor site [55,64]. It is still unclear which macrophage type is promoted by HT. However, evidence suggests that HT shifts the M2-type macrophages to the M1-type macrophages. HT is associated with the release of TNF-α and PGE2, which is associated with the M2-type macrophages [51].

#### 3.2.5. Concluding Remarks

**Figure 4:**
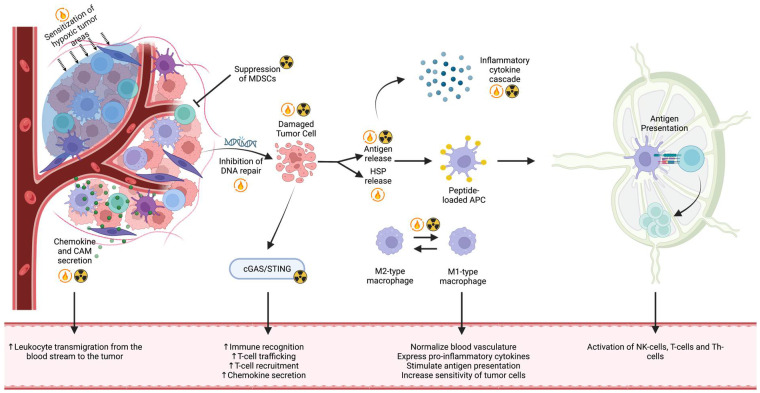
Schematic representation of the immune response induced by radiation and hyperthermia therapy. This figure illustrates the synergistic effects of hyperthermia (indicated by the little flames) and radiation therapy (indicated by the black and yellow circles) in enhancing the immune response within the tumor microenvironment and beyond. Radiation therapy activates the cGAS/STING pathway, enhancing immune recognition, T-cell trafficking, T-cell recruitment, and chemokine secretion. Radiation also normalizes blood vasculature, induces the expression of pro-inflammatory cytokines, stimulates antigen presentation, and increases the sensitivity of tumor cells to immune responses. Hyperthermia further amplifies these effects by increasing immune recognition and T-cell trafficking, as well as promoting the secretion of chemokines. The combination of hyperthermia and radiation therapy leads to an enhanced presentation of tumor antigens, which activates natural killer (NK) cells, T-cells, and helper T-cells (Th-cells). This combined approach effectively strengthens the anti-tumor immune response, providing a more robust attack on the tumor cells.

Overall, immune stimulation occurs at febrile temperatures up to 41 °C [6]. This immune stimulation involves an increased release of DAMPs, increased immune cell trafficking, and the increased cytotoxicity of T-cells and NK-cells. Figure 4 summarizes the immunological pathways of HT and RT. 

## 4. Discussion

The clinical application of hyperthermia and radiotherapy has been demonstrated in different types of cancer: head and neck cancer, melanoma, and breast, cervix, and rectal cancer [65,66,67,68,69,70,71,72]. Hyperthermia and radiotherapy share immunostimulatory and immunosuppressive complementary and synergistic immunological pathways. Synergistically, both trigger the release of danger signals, which can elicit strong systemic anti-tumor immune responses mediated by CD8^+^T-cells, causing an increase in leukocyte trafficking through the upregulation of cell adhesion molecules. The secretion of cytokines and chemokines increases the infiltration of Treg cells into the TME, and both are complementary in triggering cell death. Complementarily, HT causes sublethal damaged cell death and increased blood flow, which could result in an immune favorable TME for metastatic cells at a distance from the irradiated site. Both HT and RT upregulate extracellular tumor HSP70. Hence, we could hypothesize that, when applied together, HT and RT could significantly increase the release and amount of HSP70, causing increased DAMP recognition by macrophages and DCs. This could lead to a more powerful tumor antigen presentation in the lymph nodes, and subsequently, a more powerful immune response mediated by CTLs. Additionally, the increase in tumor cell killing observed by combining HT and RT could also lead to an enhanced release of DAMPs, as more cells are killed. Both treatment modalities also cause an increase in cell adhesion molecules. RT upregulates VCAM-1, ICAM-1 and E-selectin, whereas HT upregulates ICAM-1 and L-selectin. Again, we could hypothesize that when applied together, HT and RT could strengthen this response, leading to even stronger leukocyte adhesion at the tumor sites. This could lead to better lymphocyte trafficking into the tumor, which has the capacity to strengthen anti-tumor responses. RT and HT can potentially be more powerful when used in combination, rather than individually, due to their complementary and synergistic immunological pathways and the resulting systemic anti-tumor responses. 

We want to suggest that the most optimal protocol is combining the local RT of metastatic cancer with fever-range WBHT. Regarding this setting, this manuscript shows strong evidence that WBHT increases the chances of RT triggering systemic anti-tumor immunity and elicit abscopal effects. While RT, locally, elicits an immune reaction through the release of DAMPs—also upregulated by HT—and the activation of the cGAS/STING pathway, among others, WBHT enhances lymphocyte trafficking. This enhancement can exert its role locally and systemically. Locally, the recruitment of DCs and macrophages is enhanced, which promotes antigen uptake. Systemically, WBHT allows for the better trafficking of DCs to the tumor draining lymph nodes, enhances antigen presentation, and creates an immune favorable TME for the activated CTLs in the distant metastases. We would also like to suggest that the patient is submitted to HT right before and after RT. Delivering HT before RT could lead to an intracellular accumulation of HSPs and tumor antigens, which would be released upon subsequent RT, causing necrotic cell death. An increased release of TAAs could then be observed, which may elicit stronger antigen uptake and presentation. Applying HT after RT would cause the cell death of the sublethal damaged cells and increase immune cell trafficking. 

The resting state of tumors remains strongly immunosuppressive. Many aspects regarding the effect of HT on the recruitment and activation of M2-type macrophages, MDSCs, and TGF-ß remain unclear. Despite these challenges, HT’s ability to enhance antigen presentation and lymphocyte trafficking is promising. Therefore, exploring the combination of HT and RT holds significant potential for future breakthroughs in converting the tumor microenvironment to an immunostimulatory state. Further studies are warranted to fully uncover the immunological potential of combined HT and RT, paving the way for innovative cancer therapies.

## 5. Conclusions

Theoretically, HT and RT share complementary and synergistic immunological pathways. They both stimulate the release of DAMPs, increase lymphocyte trafficking, and complement each other in triggering cell death. HT delivery enhances the potential of RT to elicit a systemic anti-tumor immune response and trigger abscopal effects. Although the tumor and its TME remain highly immunosuppressive due to the secretion of TGF-ß and the presence of MDSCs, M2-type macrophages, and Tregs, the combined approach shows promise. Therefore, exploring the combination of HT and RT to determine its ability to convert the immunosuppressive TME into an immunostimulatory one is crucial. Further studies are warranted to confirm the immunological potential of combined HT and RT, offering hope for innovative cancer treatments.

## Data Availability

No new data were created or analyzed in this study. Data sharing is not applicable to this article.
